# A co-design process to develop personalized mobility programming for individuals with mobility impairments

**DOI:** 10.3389/fresc.2024.1336549

**Published:** 2024-11-20

**Authors:** Stephanie R. Cimino, Olivia Crozier, Daniel Lizotte, Adnan Shabbir, Joshua Stoikos, Dalton L. Wolfe

**Affiliations:** ^1^ Parkwood Institute Research, Lawson Research Institute, St. Joseph's Health Care London, London, ON, Canada; ^2^ Health and Rehabilitation Sciences, Faculty of Health Sciences, Western University, London, ON, Canada; ^3^ School of Occupational Therapy, Faculty of Health Sciences, Western University, London, ON, Canada; ^4^ Department of Epidemiology and Biostatistics, Schulich School of Medicine and Dentistry, Western University, London, ON, Canada; ^5^ Department of Computer Science, Faculty of Science, Western University, London, ON, Canada; ^6^ School of Kinesiology, Faculty of Health Sciences, Western University, London, ON, Canada; ^7^ School of Physical Therapy, Faculty of Health Sciences, Western University, London, ON, Canada; ^8^ School of Health Studies, Faculty of Health Sciences, Western University, London, ON, Canada

**Keywords:** adaptive intervention design, co-design, integrated knowledge translation, mobility, neurological conditions

## Abstract

**Introduction:**

Individuals with neurological conditions (e.g., stroke, spinal cord injury, multiple sclerosis) may experience challenges to their mobility. While the individual needs for persons with neurological conditions may vary, thus making intervention development more difficult, identifying key personalization or tailoring variables may help to customize interventions. However, the process to personalize treatments has not been well described. It is also unclear how adaptive intervention design includes the perspective of those with lived experience. Co-design methods may be a way to be transparent about intervention development to meet the needs of persons with mobility impairments while ensuring the resulting intervention is relevant and applicable to those who will be participating. The purpose of the present article is to describe a co-design process to facilitate the development of personalized mobility programming for persons with mobility impairments.

**Methods:**

Development of a set of personalized mobility programming for individuals with mobility impairments was conducted following an adaptive intervention design approach with a co-design component. A series of working groups and individual sessions with key interest groups (e.g., persons with lived experience, fitness instructors, front-line clinicians, students) were conducted in order to develop the personalized mobility programming based on the needs and preferences described during various working groups.

**Results:**

Two sets of working groups and three individual one-to-one sessions were conducted with a total of 14 participants (*n* = 6 persons with lived experience, *n* = 4 research team members, *n* = 2 physiotherapists, *n* = 2 occupational therapists, *n* = 1 registered kinesiologist). From the information gathered during the working groups a set of four personalized mobility programs were developed: (1) cognitive cardio class, (2) functional strength class, (3) mobility circuit group, and (4) an open gym. Participants also discussed the onboarding process, how to effectively track participant goals throughout the programming and personalization variables.

**Discussion:**

The current paper provides a guideline for future work that aims to develop programming that is personalized to the needs of the persons with mobility impairments due to various neurological conditions. The strengths of this approach include the collaborative nature of the program development, while the main limitations were logistical in nature (e.g., scheduling, engaging all working group members).

## 1 Introduction

Individuals with neurological conditions (e.g., stroke, spinal cord injury, multiple sclerosis) may experience a variety of health concerns, including issues with mobility. The mobility needs of this population require specific programs to address mobility related concerns. However, the needs of individuals within this population can vary greatly, which makes it challenging to create a single intervention that meets the unique needs of each participant. One approach is to identify *personalization* or *tailoring* variables, which are participant attributes that are used to customize the intervention. The process through which these personalization or tailoring variables are developed in practice is not well understood. A recent systematic review by Malmartel et al., 2021 (1) aimed to classify the methods that have been used to personalize participative interventions in randomized controlled trials. With respect to personalization specifically, the authors identified that 72% of protocols that met their inclusion criteria failed to adequately describe what tailoring variables were used in the included interventions (1). The lack of transparency throughout the currently available literature makes the development of future trials more difficult and calls into question the validity and applicability of resulting interventions for their target populations. One approach that may help to provide a transparent process while maintaining clinical significance is an adaptive intervention design.

Sequential, Multiple-Assignment Randomized Trials (SMARTs) support the development and evaluation of a sequential, individualized, multicomponent intervention which accounts for the changing needs of participants over time (2, 3). What is unique about this type of adaptive intervention is the consideration of multiple decision points over time, which are meant to tailor and individualize the intervention (2). There are four main components of adaptive intervention design: decision points, tailoring variables, intervention options, and decision rules (2). Traditionally, a treatment package method is used to develop these multicomponent interventions, followed by a randomized controlled trial to evaluate the performance of the intervention (2). However, this process does not allow for the investigation of the performance of the specific components of the treatment package (2). In order to ensure that each of the components is effective, the Multiphase Optimization Strategy (MOST) framework has been suggested (2). This framework is used to optimize and evaluate multicomponent interventions (2). MOST has three phases: (1) Preparation, (2) Optimization and (3) Evaluation. In the Preparation phase, information is gathered from currently available literature, clinical experience and other sources to develop a theoretical model (2). In addition to the development of the model, the Preparation phase is where the optimization criteria is selected (2). In the Optimization phase, decisions regarding which components meet the optimization criteria are made. Finally, the resulting intervention is assessed during the Evaluation phase (2). Unfortunately, studies that utilize the MOST framework often fail to describe the optimization and preparation phases. For example, a recent systematic review found that of the 58 articles that indicated they used the MOST Framework, there was considerable variability in how the other elements of the MOST framework such as the preparation stage were described (4).

While the MOST framework describes an important process that can help enhance intervention science, it is unclear about how persons with lived experience (PWLE) should be involved in its activities. There is substantial work in the field of integrated knowledge translation (iKT) that highlights the importance of collaborations with PWLE and other key interest groups (e.g., researchers, clinicians, representatives from SCI/D community organizations and funding agencies). One primary example is the work in spinal cord injury/dysfunction (SCI/D). The iKT guiding principles were developed by a multidisciplinary group of key interest groups described above to ensure that SCI/D related research is relevant, useful, useable, and avoids tokenism (5). Tokenism is defined as when the research users are asked to join a research project, but have little control or involvement in its construction (6). The work of this group includes strategies that fall into six categories: (1) resources and time; (2) engagement strategies in the research process; (3) communication activities and methods; (4) initiative for collaborative meetings, conferences, and/or events; (5) partnership initiation and representation; (6) education and training (6). It is through these strategies that researchers can avoid tokenism, and ensure the products are applicable to the target population.

Taken together, an adaptive intervention design that integrates the iKT guiding principles can help to ensure that meaningful engagement for key interest groups in the development of personalized mobility programming (PMP). This is what is known as a co-design approach. Co-design uses a pragmatic and inclusive research strategy, where PWLE work directly alongside researchers, clinicians, trainees and data scientists to provide iterative progress toward intervention and trial development (7). From an adaptive intervention design perspective, adopting a co-design approach can facilitate the development of a set of mobility programming by ensuring that the resulting programs are relevant to the target population. Furthermore, key tailoring variables that are required to personalize the intervention to the individual participants can be developed due to the involvement of all key interest groups (e.g., PWLE, researchers, students, fitness instructors, clinicians). The co-design process typically involves qualitative approaches (e.g., interviews or focus groups) and the formation of a working group, to ensure that the experiences of the stakeholders are captured (8–13). While it is well understood that capturing the experiences of PWLE and other key interest groups is vitally important to developing high quality, relevant and ethical research, the actual involvement of this group outside of “traditional” qualitative work in the development process is seldom described in detail. Therefore, it is the purpose of this paper to describe the co-design process undertaken during the Preparation and Optimization phase (hereby known as pre-research activities) to develop a set of PMPs and the tailoring variables using an adaptive intervention design approach. For the purposes of this work, the intervention will be the resulting PMPs.

## 2 Methods

Prior to the main research trial, we embarked on a developmental process utilizing a co-design approach. In order to develop the interventions, the MOST framework was followed (2). The Preparation phase of the project involved three formal information gathering activities: (1) environmental scan (e-scan) of current available programs; (2) interviews with PWLE regarding their involvement in mobility programming; and (3) interviews with clinicians and fitness instructors who have participated in the development or running of mobility programming. The findings from these activities will be described in future publications. This phase also involved a co-design process to inform a set of PMPs, which included determining the key decision points required to tailor the programs to meet individual needs (e.g., criteria to be eligible for participation, criteria for modifications, etc.). For the purposes of this paper, the process is described in a linear fashion, however, information gathering activities happened concurrently with program development and trial design activities. According to institutional guidelines, this was deemed a quality assurance initiative, therefore formal ethical approval was not required.

### 2.1 Co-design process

The development of the PMP was facilitated by the involvement of key interest groups including PWLE, fitness instructors, clinicians, researchers and students. With the intention of incorporating feedback of these key interest groups throughout the project, two working groups were set up that were facilitated by research staff. The first working group consisted of PWLE, fitness instructors, researchers and students. The purpose of this group was to develop ideas that could be translated into mobility programming to inform a pilot trial as part of the Preparation phase of the MOST framework (pilot trial will be described in future publications). In addition to program development, the first working group was responsible for identifying key decision points that would be used to determine which programs participants would be involved in and how those programs could be personalized to meet participants needs. The second working group consisted of clinicians working in the rehabilitation hospital (i.e., occupational therapists and physiotherapists). The second working group was asked about the developed programs including their feasibility and specific tailoring variables from the clinical perspective. In these ways, the co-design process helped to ensure that the two key components of adaptive intervention design were met (developing feasible and evidence-based programming and identifying key tailoring variables). In addition to the working groups, three one-to-one sessions were held, two with an additional clinical staff member and one with an individual with lived experience to review the PMPs that were developed.

In alignment with the iKT principles “RECIPE”, we utilized a number of strategies to ensure meaningful engagement (14). The following are examples of strategies undertaken: (1) *Resource & Time (R)*: Working groups were held virtually or in-person depending on preference; (2) *Engagement strategies in the research process (E)*: Working group members were asked to provide feedback at all stages of the development process, including the development of the interventions. Working group members were asked to provide feedback during sample classes in order to ensure that the research team captured the key aspects of programming that they working described as important; (3) *Communication activities and methods (C)*: Emails were sent to all working group participants following the sessions with the meeting notes. Working group members were asked to review the notes and provide any feedback on what was discussed during the meeting if they did not feel comfortable in the group setting. (4) *Initiative for collaborative meetings, conferences, and/or events (I)*: In addition to being able to email feedback if working group members were comfortable in the meeting, the facilitators of the working group aimed to ensure everyone had the opportunity to comment before moving on to the next question. This included directly asking specific members if they had anything to add. Furthermore, during the analysis of the data, we treated all ideas equally, ensuring that the loudest voice did not drive the development of programming; (5) *Partnership initiation and representation (P)*: Individuals were asked to participate in our working group if they had previously participated in mobility related programming at our institution. An initial meeting to discuss what the working group would entail, frequency and expectations was conducted with each potential group member. We aimed to ensure representation for a number of key interest groups including PWLE, clinicians, fitness instructors and research staff; (6) *Education and Training (E)*: Prior to the first working group, members were provided with information regarding what mobility programming was already available at our institution, to help facilitate discussions about what was missing.

#### 2.1.1 Working Group Members

Individuals with experience in either participating in or facilitating mobility programming at a large research hospital in an urban area were invited to join the first working group. The members of the working group consisted of five PWLE (e.g., persons with multiple sclerosis, spinal cord injury, brain injury), five fitness instructors, and four research team members (e.g., staff, trainees). Several of the working group members fell into multiple categories (i.e., PWLE who taught fitness programming at the rehabilitation hospital). In total, eleven individuals contributed to the first working group. To participate, individuals must have been able to join one working group session per week via Microsoft Teams.

For the second working group of clinicians, members must have had experience administering or developing mobility programming. Four individuals who were working as clinical staff at a large rehabilitation hospital participated in the second working group (*n* = 2 occupational therapists, *n* = 2 physiotherapists), and a registered kinesiologist and PWLE (i.e., individual with stroke) participated in individual sessions. Members of the second working group were asked to participate in one session via Microsoft Teams.

#### 2.1.2 Structure

##### 2.1.2.1 First working group

Each session was run using a focus group format facilitated by research staff. Sessions were held virtually or using a hybrid format. Two members of the research staff were responsible for facilitating discussion, while one additional staff member was responsible for notetaking. A focal prompt (main topic of discussion) was provided to working group members, who provided their insights and thoughts while a research staff member wrote down participant ideas on a Microsoft Word document while sharing their screen. By employing a visual display and providing real-time notes, this method effectively promoted discussions among members of the working group by enabling them to observe and listen to each other's contributions. This process was adapted from Concept Mapping procedures where a focal prompt is used to develop a list of statements regarding a specific topic (15).

The first working group session involved an introduction to the working group goals, as well as an overview of the findings from the information gathering activities described above. Decisions on what would be discussed in subsequent working group sessions were decided by the leadership team (e.g., PWLE, research staff). This was an iterative process that included developing focal prompts based on the previous weeks discussion in the working group (see Table 1 for session focal prompts).

**Table 1:** Focal prompts for working group brainstorming sessions.

Session number	Focal prompt(s) for each session
1	Introduction to working group activities What components should be included in personalized mobility programming?
2	If you were a new participant, what would you like to happen during the onboarding process before you start? If you were a fitness instructor starting a new program, what information about the participants would be most useful to have prior to beginning?
3	What programs, outside of what is already available at the rehabilitation hospital, would you like to participate in?
4	How would you like to be able to gather information about programs at the rehabilitation hospital?
5	Program trial #1 – Cognitive cardio
6	Program trial #2 – Functional strength training
7	What would the triage process look like when wanting to join one of the identified programs?
8	What would the check-in/feedback process look like for each of the identified programs?

##### 2.1.2.2 Second working group & one-to-one sessions

In the second working group, research staff introduced the clinicians to the programs that were developed (i.e., circuit group, cognitive cardio, functional strength training and open gym) and asked about the feasibility of the programming. The clinicians were also asked about key decision points for inclusion/exclusion in each of the programs, when modifications would be needed and when participation in a program should be stopped.

Two sessions were held with a clinician who was unable to attend the first or second working group sessions. The first session was a one-on-one meeting where the individual was provided with a high-level overview of the research conducted to date and an introduction to the prompts given to the working group members. The second meeting included undergraduate students who provided additional questions and prompts as needed. In this meeting, the individual was asked questions pertaining to PMP structure, key decision points and considerations for developing a novel triage system, potential exercises and activities that could be implemented in the program, the advantages and disadvantages of virtual and in-person delivery models, and the feasibility of programming for the patient populations and participants they work with. In the one-to-one session with the PWLE facilitated by the core research team, the member was asked about their thoughts on the triage process and the developed programs.

Following each of these sessions, the core research team and undergraduate trainees analyzed and implemented the feedback provided by each of the groups into trial documents and processes.

#### 2.1.3 Program development via co-design

The programs and associated tailoring variables were developed based on the information gathered during the working group sessions as well as information from the information gathering activities (e.g., qualitative interviews, e-scan). Following a review of the first four brainstorming sessions, a list of potential programs was created (e.g., functional strength training, cardio). In addition to the development of brand new programming, the current programs available at the rehabilitation hospital were also reviewed and included if they met the needs described by the working group. Members of the research team with expertise in mobility program development worked to create the programs to be used in the pilot trial (described elsewhere). In order to test the programs developed by the research team, a trial class was conducted with the working group. The trial classes consisted of an introduction to the class, including a description of the purpose, a 10–15 min trial, followed by explanations about what the full class would consist of. Following the trial, the working group was asked about their thoughts about the program. This included if the group felt that the class was representative of previous discussions, if any modifications to the program were needed, as well as what tailoring information specific to the class were needed (i.e., what should the inclusion/exclusion criteria be for the class, how can participants provide feedback, when to provide feedback, how often they wanted to provide feedback, and when they think the class should be modified on an individual level). The information from these sessions was used to create specific and comprehensive programming to be used in the next phase of the study (i.e., the pilot trial).

### 2.2 Student involvement and experiential learning

The experiential learning model used in the current study integrates the principles of iKT and co-design through the integration of trainees (e.g., undergraduate students, graduate students) from various academic backgrounds with complementary skillsets into research activities. Including trainees throughout the project enhances the co-design process by providing additional avenues of development via innovative solutions and enhanced learning. Meaningful engagement by trainees was a key aspect of the co-design process for this project, as it fostered a pragmatic and inclusive approach to the development of the PMP.

A core team composed of a post-doctoral fellow and two graduate students from professional programs (i.e., occupational therapy and physical therapy) worked collaboratively with undergraduate trainees. Six undergraduate trainees with varying skills and experiences from various disciplines (i.e., kinesiology, health sciences, medical sciences) were involved in the co-design process. In collaboration with the undergraduate trainees, the core team worked to advance the development of PMP and tailoring variables. To achieve this goal, the core group facilitated trial development activities and mentored undergraduate trainees throughout the various sub-projects associated with the overall research study (i.e., information gathering activities, working group sessions) as well as through the development of mobility programming.

In addition to the working group sessions described above, the undergraduate trainees involved in the project attended weekly check-in meetings facilitated by the core team to discuss pertinent agenda items related to ongoing trial activities, provide updates on overall project progress, and communicate any concerns about their tasks. Undergraduate trainees were assigned tasks and activities that aligned with their personal and professional goals and interests, as permitted by their capacities. Two of the undergraduate students experienced in creating and leading mobility programs helped develop the virtual Cognitive Cardio and Strength programs for the future phase of the project.

## 3 Results

Eight working group meetings were conducted with the first working group two sessions with the second working group and three one-to-one sessions, for a total of 13 sessions. This series of working group sessions resulted in the development of four mobility programs. Each working group session lasted between 30 min and 1.5 h. A brief summary of each of the first working group sessions is provided in Table 2. Information gathered from the second working group sessions and one-to-one sessions have been described below.

**Table 2:** Brief overview of the first working group discussions by session.

Session number	Summary of discussions
1	Introduction to working group activities, purpose and goals of the group.
2	Participants in the working group spoke about logistical considerations such as virtual or in-person, whether instructors should be able-bodied and if that was the case, should they be accompanied by a PWLE. The inclusion of one-on-one time with instructors was also discussed. The group also began preliminary discussions about content, which included needing to have fun and enjoyable programming, how to ensure the programming was fun (e.g., via check-ins) as well as having a variety of options for people to chose from.
3	Both PWLE and the fitness instructors expressed interest in wanting to have a meeting one-on-one prior to classes beginning so the PWLE could share information about their condition and limitations. From the instructor perspective, instructors would like to get to know participants so that they can make modifications to the class and help them feel more prepared. With respect to the content of the onboarding process, PWLE talked about wanting to know about any potential barriers there may be to participating in the program. They felt strongly about getting to talk with instructors prior to beginning any program. Fitness instructors would prefer one-on-one sessions (like a trial class) so that potential participants could flag what worked and what didn't work throughout the class. Logistics were also discussed, particularly if the onboarding should be mandatory, how far in advance the process should start and to decide which on-boarding process would be best for the participant.
4	Participants discussed having levels of classes (e.g., beginner/more advanced) so people don't get discouraged. There was a lot of emphasis of having programs consider things that weren't necessarily physical like mental well-being. Working group members wanted to incorporate something to help with skills to alleviate and regular stress. The working group felt strongly about developing some sort of pool program to help people gain confidence in the water. Other suggestions for programming included a functional skills class, a general exercise literacy class, open gym/circuit training course, and ensure that some sort of social aspect incorporated. The social aspect was important in order to provide mental support and destress.
5	Trial cognitive cardio class. Participants generally found this to be a great program and felt that the class would meet the needs of the current project. The group discussed the logistics of having a hybrid model class (e.g., in person and online options), as well as asynchronous options (e.g., videos to review if participants could not attend class during class time). With respect to personalization, participants encouraged the research team to explore other options for the cognitive portion of the class. For example, rather than having just riddles, perhaps include trivia or visual puzzles depending on the needs of the participants to be determined at the beginning of the class.
6	Trial functional strength class. Working group participants felt that this class would meet the needs of a personalized mobility program and like the overall design of the class. The group felt that having different levels of classes would work well for this type of training with options to have asynchronous videos available. When asked about making this type of class a companion class to an in-person circuit style mobility training program, participants felt that this would exclude those who could not attend in-person. Logistics of hybrid style were discussed, and the group described the difficulties of using a hybrid style as it may isolate individuals who were joining virtually. Decisions around keeping the class entirely virtual were made to accommodate the majority of individuals. Preliminary discussions about onboarding and check-in modalities were initiated.
7	During this session, participants described liking the idea of having to complete a survey about their demographics and preferences prior to enrolling in a specific program. Participants also described wanting the option to have an open gym where they were not restricted by needed to attend specific class times that may not work with everyone's schedule. Logistics of the trial were also discussed (e.g., self-referral to the program, how many trial participants will there be at a time, who will be running sessions, etc.).
8	Participants during this session discussed wanting to have a booklet in order to track their progress over the course of their participation in the mobility programming. At the beginning of the booklet, an initial assessment would be included which would involve SMART goal setting. The participants then envisioned that the individuals taking part in the mobility programming could reflect on their goals each week and fill out surveys for program feedback. Instructors would also fill out surveys about how they feel the program is going. Participants in the working group session also discussed what areas they considered should be measured in order to determine if a program was successful. High level domains included quality of life, goal attainment, self-esteem, self-efficacy, and social connectedness.

### 3.1 Types of programs developed

Based on the information provided throughout the working group sessions, four main mobility programs were developed. Two programs were adapted based on already available programs at the rehabilitation hospital. The first of these programs was a cognitive cardio class and the second was a functional strength class. The third program was a newly developed program, which will include a circuit style mobility training class for those with various mobility or functional skill goals. The fourth program that will be made available is an open gym format, where participants can come in and use various equipment and complete their own workouts (see Table 3 for descriptions of the PMPs).

**Table 3:** Descriptions of personalized mobility programming.

Name of program	Method of program delivery	Description of program
Cognitive Cardio	Virtual	Cognitive Cardio is a moderate- to high-intensity class with an emphasis on cardio. This class involves answering two riddles and one trivia question while completing a cardio exercise (e.g., marching, seated or standing). For each riddle/trivia the participant will have a 45-second buffer before they can shout out the answer so that everyone can have a chance to think it through. After 3 riddles/trivia have been answered, there will be a break before moving on to the next exercise. There will be a total of five exercises with two riddles and one trivia question each. This class offers variations that incorporate lower body movement for those who are interested, and all exercises are modifiable to meet the level of physical function of participants. Equipment is not needed for this class and music is not played in this class to allow less distractions while thinking about the riddles.
Strength Class	Virtual	Strength Building aims to program that focuses on building strength to make it easier to complete everyday tasks and lower the risk of injury. This class focuses on improving upper and lower body and core strength. The class runs for 60 min once a week for six weeks. The class takes place online. Music is not played in this class. Each class will begin with a warm-up, followed by rehearsal moves that will go over the exercises that will be completed during the class. Exercises will be chosen based on participants goals and to introduce participants to new exercises that they will get a chance to practice bi-weekly. Participants will alternate between two routines that will be completed every other week.
Mobility Circuit Group	In-person	Mobility Skills Circuit Group is a skills-based program that focuses on improving one's ability to move more freely and easily, specifically learning skills to enhance your use of a mobility aid, transfers, endurance, and activities of daily living/instrumental activities of daily living. Our mobility influences our ability to do the activities of daily living that we enjoy. This class focuses on improving range of motion, balance, coordination, fine motor skills, endurance, gait, standing, walking, transfers and self-care. The class runs once a week for six weeks. The class is completed in-person in a small group. Music is not played in this class. There is a 90-minute social component. Each class will begin with a warm-up, followed by four stations (mobility, transfers, endurance, and activities of daily living/instrumental activities of daily living (ADLs/IADLs).
Open Gym	In-person	Open Gym offers a dynamic, self-guided fitness experience suitable for individuals of all mobility and strength levels. This program benefits those familiar with exercise equipment and workout routines. Beginners are not left behind, as our skilled student trainers can provide comprehensive introductions to gym equipment. Under the watchful eye of these trainers, you can progress at a comfortable pace, focusing on personal fitness objectives such as enhancing strength, boosting endurance, or improving flexibility. Whether you prefer to exercise independently, seek guidance, or engage with fellow fitness enthusiasts, our gym fosters a welcoming environment for all. We actively encourage social interactions among participants, creating a vibrant community atmosphere that enhances the overall workout experience.

### 3.2 Key aspects of personalization

With respects to logistics of the programs, participants in the first working group discussed having as many options available as possible. This included a mix of virtual and in-person classes, as well as the availability of asynchronous options. The timing of the classes was also discussed by the first working group, as individuals with different needs who would be attending the group would likely require different times (e.g., morning routines may be lengthy, younger individuals may be working and can't attend class during the day).

#### 3.2.1 Onboarding

**Figure 1:**
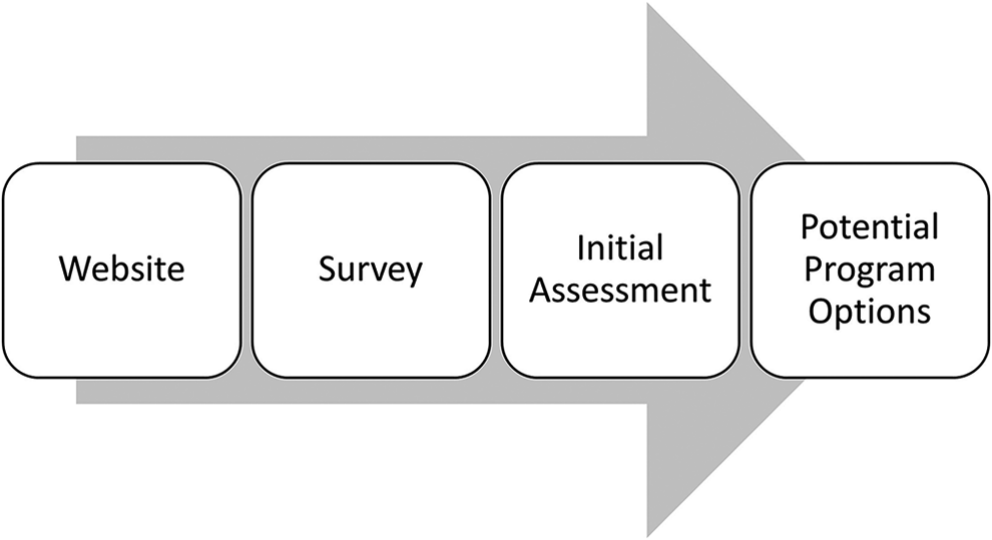
Onboarding process. (1) Website: potential participants will be directed to a website where they can review the available programming. Written descriptions and video introductions will be available for individuals to review. (2) Survey: if the individual is interested, they will be asked to complete a short survey in order to better understand their needs and goals. (3) Initial assessment: The research team will review the answers of the survey and come up with a set of programming options. The research team will meet with the interested individual to go over their responses and discuss their goals and mobility needs more in depth. (4) Potential program options: During the session, the research team member will discuss the potential programming options with the individual and determine which programs will best meet their needs. Once the individual is happy with the selected programs, they will be enrolled in the 6 week program.

The onboarding process was extensively discussed by working group members. The resulting process can be found in Figure 1. Participants in the first working group described being able to self-refer to the mobility programs, but clinicians in the second working group felt that individuals should be medically cleared to participate. One of the key topics of discussion during this session was whether the individuals participating in the program required an attendant. The need for an attendant was identified as a key consideration by clinicians for inclusion or exclusion in the mobility programming. In addition to the need for an attendant, clinicians also discussed that falls risk should be a factor when deciding if the program was appropriate for a participant. Other criteria for exclusion included whether a potential participant was able to accurately determine their limitations (e.g., lack of awareness of their challenges due to cognitive issues). Furthermore, if the participant could not determine their limitations on their own, they would require an attendant to remind them of their capabilities. This aligns with the clinicians thoughts about the process to enter the mobility programming, where the working group members described needing a referral to the program by a clinical team member.

As part of the on-boarding process, members in both working groups discussed using a survey to gather specific information about the participants who would be joining the mobility programming. Key aspects of the survey highlighted by both working groups and during the one-to-one sessions included information about participant's health conditions, communication preferences and importantly, their program preferences. Members in the first working group communicated that trying to fit the program to the participants preferences such as music or no music, group size, goals, amongst others, would be a key component of ensuring the program was personalized to the participant.

Based on the feedback of the first working group, the development of a booklet would be used from the beginning of the program and would be used throughout their involvement. At the beginning of the booklet, information gathered during the initial assessment would be included to track progress throughout the program. Members of the first working group also spoke strongly about having one-on-one sessions with instructors prior to the beginning of the program to ensure that their safety concerns would be met. This was the basis for the decision about including an initial assessment.

#### 3.2.2 Within program tailoring

Several working group sessions revolved around the participants of a personalized mobility program ability to provide feedback to the instructors throughout the program. Discussions included when to provide feedback and how to provide feedback. The modality of feedback provisions was dependent on the type of program being delivered. For example, during a low impact class such as the cognitive cardio class, the working group members did not feel as though weekly feedback was necessary. Instead, a possible option would be to have participants fill out a survey about how the program is going and if participants felt they were not meeting their goals or were having problems this would be flagged to the instructors for follow-up.

When asked about the structure for program reflection, the clinicians in the second working group discussed several key considerations when curating resources for the booklet described by the first working group. Clinicians described using Likert type scales or symbols instead of open spaces for writing for those who may have trouble with writing, offering both hard copy and digital formats for those who may use assistive technology as well as interpreters where available. Thoughts on resources for the booklet included an education section about how to set SMART goals, and a goal attainment scale such as the Goal Attainment Scaling (GAS) (16).

Decisions about when the programs should be stopped were discussed by participants in the first working group. Members from the first working group felt that participation in the mobility programming should be stopped if the participant was having physical problems participating in the exercises or the program was no longer meeting the goals of the participant.

## 4 Discussion

The co-design process that was undertaken in the current study highlights the effectiveness of using such an approach to develop PMP and related tailoring variables. This paper fills a gap in the current literature, where articles seldom describe how the personalization and tailoring process is determined (1). It is critical to be transparent about the processes used to determine personalization in order to ensure that the programming can be easily replicated (17, 18). Key take-aways from this work include how to incorporate perspectives from a broad range of stakeholder groups, including PWLE, clinicians, and researchers. This paper also provides insights on how to ensure meaningful engagement in the development of research programs (e.g., trial classes).

### 4.1 Strengths of our approach

There are a number of strengths to using the collaborative approach to adaptive intervention design described here. Our co-design process followed the well established iKT principles set out by the University of British Columbia, for integrating PWLE experience into all aspects of the research (5, 6). Previous literature suggests that interventions (which in the case of the current article is the PMP), that are developed via co-design are more likely to be to be acceptable to providers and end users, which increases the likelihood of adoption (19). To enhance the comprehensiveness of our approach and gather diverse perspectives, we recruited a wide range of individuals with different neurological conditions such as stroke, multiple sclerosis, brain injury, and spinal cord injury. Additionally, we included front line clinicians and students from various disciplines to further enrich our project. The diversity of members across our working groups and one-to-one sessions ensured that the information we gathered was representative of the context within which the PMP will be conducted. Furthermore, the use of working groups and individual sessions provided the opportunity to get direct feedback on the suggested programming and other trial components (e.g., onboarding, website design, booklet, etc.), which will help to ensure that the mobility programming to be used in the pilot trial will likely be relevant and applicable to a diverse group of participants with varying mobility needs.

Also unique to our approach is the involvement of students and the experiential learning. Involving students from various disciplines not only provided them with an opportunity to put their theoretical knowledge into practice, but provided our team invaluable perspectives on the development of the different aspects of the program.

### 4.2 Limitations of our approach

While we aimed to undertake as rigorous a process as possible, this approach does not come without it's limitations. The main limitation was logistical in nature, mainly involving the scheduling of the working groups. First, given the schedules of clinicians, we were unable to have them attend the working group sessions with the PWLE and fitness instructors. While it would have been helpful to have the clinicians attend multiple sessions, we were able to get highly relevant information from the times were able to speak with them directly. Another limitation with scheduling was attempting to coordinate a relatively large number of people with different schedules, which delayed the initial start of the process. However, once we had found a time that worked on a weekly basis for the first working group members, scheduling was less of an issue. For those individuals who expressed interest in participating but were unable to join our groups, we provided additional opportunities for participation (e.g., the second working group, individual sessions with members). While this did create more work for the research team, these sessions were invaluable to the process to ensure we captured as many perspectives as possible to inform the PMP and tailoring variables.

Similar to qualitative focus groups, there are limitations of facilitating these types of groups (e.g., managing differing personalities, ensuring all participants are able to share their thoughts). While the facilitators of the working groups did their best to engage all members, it is possible that some members were unable to share their thoughts or did not feel comfortable in this type of setting. To best address this challenge, the research team offered the working group members the option to share their opinions via email after the working group sessions. In addition to this, the notes of each meeting were sent to the members and the members were encouraged to let the research team know if there was anything missing from the notes or if there was anything they would like to add.

A final limitation of this work was the lack of evaluation of meaningful engagement of our working group members. While we undertook a number of strategies to ensure that our working group members were able to participate as fulsomely as possible, there is a possibility that some participants may not have felt valued or felt their participation was not valued. Future work should aim to evaluate the strategies used in the co-design process to ensure that participants feel engaged throughout the process.

### 4.3 Implications

The current paper provides a guideline for future work that aims to develop programming that is personalized to the needs of the persons with mobility impairments due to various neurological conditions. We encourage those who are developing similar programming to be as transparent as possible about their processes for determining personalization and tailoring variables. While the current article is focused on mobility programming, the co-design process described here is likely applicable to the development of other interventions that include personalization and tailoring. Despite the limitations of using and facilitating a co-design process, the rigour that it provides to ensuring that the resulting programming is applicable and relevant to the target population is invaluable.

## 5 Conclusion

Overall, the co-design process described here resulted in an initial set of PMP for persons with varying levels of mobility impairments. This paper fills multiple gaps in the current literature, including a lack of transparency about how tailoring variables are developed and how stakeholders are included in program design. The process undertaken throughout the pre-research activities provides an example of how to promote meaningful engagement in the co-design of PMPs and associated tailoring variables. The next phase of the project will involve a pilot trial to better understand the feasibility of implementing the mobility programs developed, including the acceptability to a larger range of individuals with mobility impairments.

## Data Availability

The raw data supporting the conclusions of this article will be made available by the authors, without undue reservation.
